# Sesquiterpene Lactones from *Artemisia* Genus: Biological Activities and Methods of Analysis

**DOI:** 10.1155/2015/247685

**Published:** 2015-10-01

**Authors:** Bianca Ivanescu, Anca Miron, Andreia Corciova

**Affiliations:** ^1^Department of Pharmaceutical Botany, Faculty of Pharmacy, University of Medicine and Pharmacy “Grigore T. Popa”, 16 Universitatii Street, 700150 Iasi, Romania; ^2^Department of Pharmacognosy, Faculty of Pharmacy, University of Medicine and Pharmacy “Grigore T. Popa”, 16 Universitatii Street, 700150 Iasi, Romania; ^3^Department of Drug Analysis, Faculty of Pharmacy, University of Medicine and Pharmacy “Grigore T. Popa”, 16 Universitatii Street, 700150 Iasi, Romania

## Abstract

Sesquiterpene lactones are a large group of natural compounds, found primarily in plants of *Asteraceae* family, with over 5000 structures reported to date. Within this family, genus *Artemisia* is very well represented, having approximately 500 species characterized by the presence of eudesmanolides and guaianolides, especially highly oxygenated ones, and rarely of germacranolides. Sesquiterpene lactones exhibit a wide range of biological activities, such as antitumor, anti-inflammatory, analgesic, antiulcer, antibacterial, antifungal, antiviral, antiparasitic, and insect deterrent. Many of the biological activities are attributed to the *α*-methylene-*γ*-lactone group in their molecule which reacts through a Michael-addition with free sulfhydryl or amino groups in proteins and alkylates them. Due to the fact that most sesquiterpene lactones are thermolabile, less volatile compounds, they present no specific chromophores in the molecule and are sensitive to acidic and basic mediums, and their identification and quantification represent a difficult task for the analyst. Another problematic aspect is represented by the complexity of vegetal samples, which may contain compounds that can interfere with the analysis. Therefore, this paper proposes an overview of the methods used for the identification and quantification of sesquiterpene lactones found in *Artemisia* genus, as well as the optimal conditions for their extraction and separation.

## 1. Introduction

Sesquiterpene lactones (SLs) are probably the largest class of secondary metabolites in plants, with over 5000 structures reported to date [1–4]. They are fifteen carbon compounds formed from condensation of three isoprene units, followed by cyclization and oxidative transformation to make a* cis* or* trans*-fused lactone. The *γ*-lactone ring, usually with an *α*-methylene group, is a significant characteristic of SLs. Their molecule may present hydroxyls, esterified hydroxyls, or epoxide groups, some SLs occur in glycosylated form, and few contain halogen or sulfur atoms [5]. Sesquiterpene lactones are bitter, colourless substances, with lipophilic character and a variety of structural arrangements. They are classified depending on their carboxylic skeleton into the following main groups: germacranolides (10-membered rings), the largest group and biogenetic precursors of the majority of sesquiterpene lactones; eudesmanolides and eremophilanolides (6/6-bicyclic compounds); and guaianolides, pseudoguaianolides, and hypocretenolides (all 5/7-bicyclic compounds) [6, 7]. Sesquiterpene lactones play an important role in communication between plants and interaction with insects, microorganism, and animals acting as attractants, deterrents, and antifeedants [1, 8, 9]. One plant species usually produces one type of sesquiterpene lactones, found chiefly in leaves and flowers in concentrations of 0.01% to 8% dry weight [5, 10].

Although SLs are present in approximately 16 plant families, they are prevalent in* Asteraceae* family where they can be found in almost all genera, notably in* Artemisia*,* Arnica*,* Ambrosia*,* Helenium*,* Tanacetum*, and* Vernonia* [1, 11]. Within this family, genus* Artemisia* is very well represented having approximately 500 species, distributed worldwide and thriving in various habitats.* Artemisia* species are aromatic plants exploited for their volatile oil [12] and many of them are used all over the world in traditional medicine in order to treat conditions such as fever, malaria, inflammation, ulcer, diabetes, and intestinal worms. Morphological and phytochemical variability characterises this genus and also polyploidy is commonly reported, so different chemotypes and cytotypes will synthesize diverse metabolites [13]. SLs are produced in large amounts in glandular trichomes in response to biotic stresses but are also found in secretory canals of underground plant organs [2]. The most common SLs in* Artemisia* species are guaianolides, eudesmanolides, and germacranolides. Probably, the best known compound in this group is an endoperoxide SL isolated from* Artemisia annua*, artemisinin, a modern antimalarial used in artemisinin combination therapies that also displays anticancer activity.

The biological activity of SLs is mainly attributed to the *α*-methylene-*γ*-lactone group (*α*M*γ*L) in their structure. The *α*M*γ*L acts as a Michael acceptor and reacts with nucleophiles (sulfhydryl or amino groups) in enzymes, transcription factors, and other proteins, alkylating them irreversibly [8, 14]. The alkylation will disrupt the proper function of the biological macromolecule due to steric and chemical changes. This is considered to be the primary mechanism of action of SLs that underlies their cytotoxicity. It also explains cell wall damage in microbes and prevalence of contact dermatitis in humans. Yet, other factors can influence the potency of SLs: number of alkylating groups, lipophilicity, molecular geometry and size, chemical environment, other functional groups neighboring the *α*M*γ*L, and the target sulfhydryl [2, 5].

Considering the increasing importance of SLs from* Artemisia* genus and potential applications in medicine and agriculture, this paper aims to review the recent information relative to biological activities and analysis methods of these molecules. The knowledge of different types of analysis methods is necessary for the analyst that must choose the most appropriate method for the sample, taking into account the available equipment. The most common methods applied to SLs are chromatographic techniques, particularly HPLC with different detection methods, followed by GC. Since these methods are difficult, time consuming, and expensive, we have also chosen to present some analysis methods that are cheaper and available to all laboratories, such as spectrophotometric techniques (UV-Vis) and TLC.

## 2. Biological Activities of Sesquiterpene Lactones

### 2.1. Antitumor Activity

The antimalarial drug artemisinin and its derivatives are very potent anticancer compounds, highly selective on cancer cells with almost no side effects on normal cells and a broad spectrum of action: leukaemia, colon, melanoma, osteosarcoma, pancreas, breast, ovarian, prostate, hepatic, renal, central nervous system, and lung cancer cells [15–18]. Some disadvantages of artemisinin, such as low solubility, short plasma half-life, and poor bioavailability [19], were surpassed by the semisynthetic or fully synthetic derivatives, such as artesunate, artemether, dihydroartemisinin, and artemisone.

**Figure 1:**
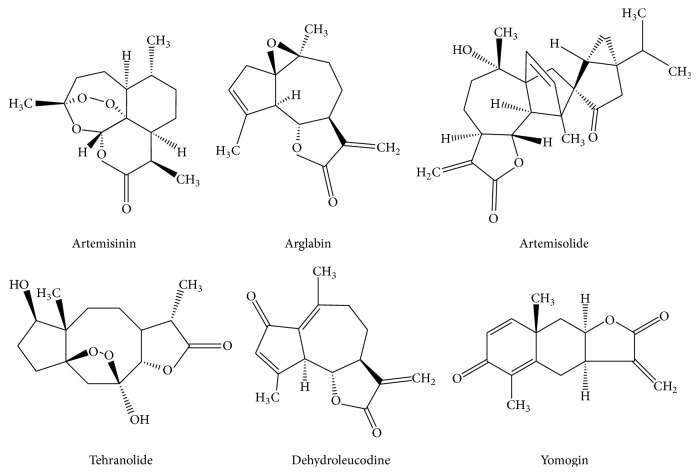
Structures of bioactive sesquiterpene lactones from* Artemisia* genus.

Artemisinin (Figure 1) is a cadinanolide with a 1,2,4-trioxane ring system, found most importantly in* Artemisia annua* L. and in minor quantities in* A. apiaceae* Hance and* A. lancea* Vaniot [20]. The presence of artemisinin in* Artemisia sieberi* and* Artemisia scoparia* in small quantities was also reported [21, 22]. However, other bioactive compounds in* Artemisia annua* contribute to the overall activity of extracts: SLs arteannuin B and artemisitene, but also scopoletin and 1,8-cineole [23]. The flavonoids present in* Artemisia annua* act synergically with artemisinin against malaria and cancer: they modify the absorption and metabolism of artemisinin in the body and exhibit beneficial immunomodulatory activity in cancer patients [24].

The antitumor mechanism of artemisinin is based on cleavage of its endoperoxide bridge by the iron in cancer cells and formation of free radicals. Free radicals will produce cell alterations such as apoptosis, deoxyribonucleic acid (DNA) damage, modulation of nuclear receptor responsiveness, arrest of growth, inhibition of angiogenesis, inhibition of tumour invasion, migration, and metastasis. These pleiotropic effects can account for effectiveness of artemisinin compounds in multidrug resistant types of cancer [25].

Some artemisinin derivatives reached the phase of clinical trials: the efficacy of artesunate combination therapy was evaluated in advanced breast cancer and another trial assessed the activity and tolerability of artesunate in colorectal adenocarcinoma [6]. A clinical trial in 120 patients with advanced non-small cell lung cancer tested the effect of artesunate in combination with traditional chemotherapeutic drugs [26]. A pilot study in ten patients with advanced cervix carcinoma proved the efficiency of dihydroartemisinin [27]. For some compounds, individual clinical cases were reported: artemether oral treatment was used in a patient with pituitary macroadenoma [28], and artesunate was used in laryngeal squamous cell carcinoma [29] and metastatic uveal melanoma [30] with good results and lack of side effects.

In the early 1980s, arglabin (Figure 1) was isolated from the Kazakhstan endemic plant* Artemisia glabella* Kar. et Kir. and was approved for use for cancer treatment in 1996 in the same country. The compound prevents farnesylation of cell proteins, killing both normal and cancer cells, with a 50–100 times increased toxicity for tumor cells [31]. Arglabin is found in all plant organs and throughout the entire period of vegetation in concentrations of 0,08–0,6% [32]. The compound was also identified in* A. myriantha* [33].

The aerial parts of* Artemisia amygdalina* Decne produce significant amounts of ludartin, a highly cytotoxic guaianolide, also found in* Artemisia indica* [34]. Ludartin displays IC_50_ values of 6.6 *μ*M and 19.0 *μ*M against mouse melanoma (B16F10) and human epidermoid carcinoma (A-431) in MTT assay [35]. Ludartin is a position isomer of arglabin and can be easily converted into clinically important antitumor arglabin [36].

Arteminolides A–D, sesquiterpene lactones extracted from the aerial parts of* Artemisia argyi,* are potent farnesyl-protein transferase (FPTase) inhibitors with IC_50_ values of 0.7–1 *μ*M. They inhibit tumor growth in mouse xenograft models and in human tumour xenograft [37]. Another cytotoxic compound produced by* Artemisia argyi* is artemisolide (Figure 1), sesquiterpene lactone with a cyclopropane ring which exhibits* in vitro* activity against human acute lymphoblastic leukaemia Molt-4, promyelocytic leukaemia HL-60, and SW620 colon cancer cell lines [38].

Yomogin (Figure 1), a eudesmane sesquiterpene lactone isolated from* Artemisia princeps,* has been shown to inhibit tumor cell proliferation [39]. Yomogin synergistically increased differentiation of human promyelocytic leukemia HL-60 cells when combined with 1,25-dihydroxyvitamin D or all-trans-retinoic acid and stimulated differentiation to monocytes, respectively, granulocytes. So, these combinations can be used in therapy of myeloid leukemias [40]. Moreover, yomogin induces apoptosis in human promyelocytic leukemia HL-60 cells through caspase-8 activation, Bid cleavage, and Bax translocation to mitochondria, followed by release of cytochrome c into the cytoplasm [41].

Eight highly oxygenated guaianolides, named artemdubolides A–H, were isolated from* Artemisia dubia* and two of them manifested reduced cytotoxicity on human colon carcinoma Colo205 and human melanoma MDA-MB-435 cells* in vitro* [42].

A new antitumor sesquiterpene lactone with an endoperoxide moiety, tehranolide (Figure 1), was isolated from* Artemisia diffusa*. Tehranolide selectively inhibits proliferation of breast cancer cells through cell cycle arrest and apoptosis [43] and also modifies the immune responses and increases antitumor immunity [44].

### 2.2. Anti-Inflammatory and Immunomodulatory Effect

SLs also exhibit anti-inflammatory and immunomodulatory actions, properties that can be beneficial in tumour treatment or chronic diseases and can enhance the success of therapy. The main mechanism of anti-inflammatory activity is by inhibiting the expression of nuclear factor *κ*B (NF-*κ*B). NF-*κ*B is a ubiquitous protein that regulates over 150 inflammatory genes and mediates immune response in humans. NF-*κ*B controls the response of other effectors such as cytokines, inflammatory molecules, and cell adhesion molecules [2]. Therefore, inhibition of NF-*κ*B decreases inflammatory response and suppresses cancer growth. In an extensive study comprising over 100 sesquiterpene lactones, researchers established that guaianolides are most potent inhibitors of NF-*κ*B and their efficacy is due mostly to the *α*,*β*-unsaturated carbonyl group [45].

Artemisinin inhibits the secretion of tumour necrosis factor (TNF)-*α*, interleukin- (IL-) 1*β*, and IL-6 in a dose-dependent manner, thus exerting an anti-inflammatory effect on phorbol myristate acetate- (PMA-) induced THP-1 human monocytes [46]. Moreover, in a mouse model of contact hypersensitivity, topical administration of artemisinin produced anti-inflammatory and immunomodulatory effects [47].

Dihydroartemisinin inhibits phorbol 12-myristate 13-acetate- (PMA-) induced COX-2 expression in murine macrophage RAW 264.7 cells via downregulation of AKT and MAPK kinase signaling pathways. Dihydroartemisinin decreased PMA-induced COX-2 expression and PGE 2 production, as well as COX-2 promoter-driven luciferase activity in a dose-dependent manner [48].

Both artemisinin and dihydroartemisinin suppress delayed hypersensitivity to sheep blood cells in mice, manifesting immunosuppressive action [49, 50]. Dihydroartemisinin also impaired growth of ductal carcinoma in mice and decreased the levels of interleukin IL-4 [50]. Artemisinin diminishes the number of regulatory T cells in murine breast cancer model [51].

Artesunate is therapeutically relevant to inflammatory responses of microglial cells [52] and inhibits production of interleukin IL-1*β*, IL-6, and IL-8 in human rheumatoid arthritis through NF-*κ*B inhibition [53].

SLs artemisinin, dihydroartemisinin, artemisinic acid, and arteannuin B significantly reduce LPS-activated production of prostaglandin E2 (PGE2). Arteannuin B also inhibited lipopolysaccharide- (LPS-) induced* in vitro* production of nitric oxide (NO) and secretion of cytokines (VEGF, IL-1*b*, IL-6, and TNF-*α*) [54].

One study evaluated the enriched sesquiterpene lactone fraction from* Artemisia annua* on different nociceptive and inflammatory animal models. The sesquiterpene lactones fraction containing artemisinin (1.72%) and deoxyartemisinin (0.31%) demonstrated pain relief on chemical-induced nociception assays in mice. The i.p. treatment produced a relevant reduction in the reaction time of the animals in both phases of the formalin test, significantly reduced the sensitivity to mechanical allodynia stimulus, reduced the paw edema caused by carrageenan injection, and promoted high antinociceptive activity in tail flick model suggesting relationship with the opioid system [55].

Another NF-*κ*B inhibitor, artemisolide, was isolated from* Artemisia asiatica* by activity-guided fractionation using the NF-*κ*B mediated reporter gene assay [56, 57]. Artemisolide suppresses production of prostaglandin E_2_ and nitric oxide (NO) in macrophages. In the same way, other bioactive sesquiterpene lactones were isolated from* Artemisia sylvatica*: arteminolides B and D, moxartenolide, deacetyllaurebiolide, 3*α*,4*α*-epoxyrupicolins C–E, and 3-methoxytanapartholide. All separated compounds also inhibited NO and TNF-*α* production [58].

Nitric oxide (NO) is synthesized in the body through oxidation of L-arginine by a family of synthases that can be constitutive (cNOS) or inducible (iNOS). iNOS induction in tissues increases the concentration of NO and can cause inflammatory effects including vasodilation, edema, and cytotoxicity. The induction of the enzyme is mediated by proinflammatory cytokines such as *γ*-interferon, tumor necrosis factor (TNF), IL-1, and IL-6. Thus, iNOS enzyme has become a new target for pharmacological research to find new substances useful in the treatment of chronic inflammatory diseases.

The anti-inflammatory effect of dehydroleucodine (Figure 1) isolated from* A. douglasiana* was investigated in arthritis induced by Freund's adjuvant carrageenan-induced and cotton pellet-induced granuloma. Dehydroleucodine inhibited both chronic and acute carrageenan-induced inflammations but was most efficient in the chronic phase. The sesquiterpene lactone also inhibited inflammation in the granuloma test, probably by interfering with transcription factors, such as NF-*κ*B and cytokines [59].

Yomogin, an eudesmane sesquiterpene isolated from* Artemisia princeps*, exhibits intense anti-inflammatory activity. It has been shown that yomogin inhibits NO production in LPS-activated RAW 264.7 cells by suppressing i-NOS enzyme expression [60] and blocks the degranulation of mast cells by inhibiting the release of beta-hexosaminidase from the cultured RBL-2H3 cells in a dose-dependent manner [61]. Also, yomogin exhibited a novel histamine H_1_ receptor antagonism in the guinea pig ileum [62].

Arglabin, a sesquiterpene lactone isolated from* Artemisia myriantha* Wall, manifests immunomodulating properties. Arglabin triggered the production of cytokines involved in host defence mechanisms: IL-1, TNF-alpha, and IL-2. Lower concentrations of arglabin were the most effective in inducing cytokines secretion [63]. Furthermore, arglabin exhibits antiexudative and antiproliferative properties on the models of acute inflammation caused by formalin, carrageenan, and histamine and on the model of proliferative inflammation accompanying cotton-pellet granuloma [64]. It has been shown that arglabin effectively attenuates the high glucose-stimulated activation of NF-*κ*B, the degradation of I*κ*B*α*, and the expression of MCP-1, TGF-*β*1, and FN in rat mesangial cells [65]. A recent study proposes that arglabin could be a promising new drug to treat inflammation and atherosclerosis, based on its pharmacological actions: it reduces inflammation and plasma lipids, increases autophagy, and orients tissue macrophages into an anti-inflammatory phenotype in ApoE2.Ki mice fed a high-fat diet [66].

Other anti-inflammatory sesquiterpene lactones mentioned in the literature are dimeric guaianolides from* Artemisia anomala* [67], and those isolated from* Artemisia khorassanica* Podl., which inhibits iNOS and COX-2 expression through the inactivation of NF-*κ*B [68]. SLs barrelierin, artemalin, barrelin, and desoxyvulgarin from* Artemisia barrelieri* also exhibited anti-inflammatory activities [69].

### 2.3. Antiulcer Activity

Sesquiterpene lactones of the guaianolide and eudesmanolide types are considered to be of interest in treatment of gastric and peptic ulcers because they have an effect in the regulation and prevention of oxidative damage and inflammation-mediated biological damage [70]. Dehydroleucodine, a sesquiterpene lactone isolated from the aerial parts of* Artemisia douglasiana* Besser, exerts* in vivo* cytoprotective actions against ethanol-induced gastric mucosal injury.

Several related guaianolides and pseudoguaianolides were also found to exhibit cytoprotection: ludartin, 8-angeloyloxy-3-hydroxyguaia-3(15),10(14),11(13)-trien-6,12-olide, hymenin, mexicanin I, helenanin, and 9-O-desacetylsparthulin-2-O-angelate. Desacetoxymatricarin did not show cytoprotective activity, suggesting that the presence of the alpha-methylene-gamma-lactone moiety is a requirement for the antiulcerogenic activity [71].

Dehydroleucodine exhibits anti-inflammatory and gastrointestinal cytoprotective action [59]. The compound stimulates mucus production and inhibits histamine and serotonin release from intestinal mast cells [72] and could act as a selective mast cell stabilizer by releasing cytoprotective factors and inhibiting proinflammatory mast cell mediators. Gastrointestinal mast cells are involved in pathologic effects but also play a protective role in defense against parasitic and microbial infections. Thus, it is believed that stabilization of mast cells may be a key mechanism in the protection of gastrointestinal tract from injury [73, 74].

The crude ethanol extract and the enriched sesquiterpene lactone fraction of* Artemisia annua* aerial parts exhibited antiulcerogenic activity on the indomethacin induced ulcer in rats. The sesquiterpene lactone fraction yielded three different polarity fractions on column chromatography. For the medium polarity fraction, it was demonstrated that the active compounds of* Artemisia annua* act by increasing the prostaglandin levels in the gastric mucosa [75].

Three SLs isolated from the ethanol extract of* Artemisia annua*—artemisinin, dihydro-epideoxyarteannuin B, and deoxyartemisinin—were tested on ethanol and indomethacin-induced ulcers in rats. Both dihydro-epideoxyarteannuin B and deoxyartemisinin reduced the ulcerative lesion index produced by ethanol and indomethacin, while artemisinin did not manifest cytoprotection. Previous treatment with indomethacin, a cyclooxygenase inhibitor, blocked the antiulcerogenic activity of compounds on ethanol-induced ulcer, suggesting that the activity is the consequence of an increase in prostaglandin synthesis [76].

Furthermore, SLs may exhibit another benefic effect in ulcer through their antimicrobial activity. Thus, artemisinin and its analogues manifested remarkably strong activity against* Helicobacter pylori*, the pathogen responsible for peptic ulcer diseases [77]. Both dehydroleucodine and* Artemisia douglasiana* extract showed* in vitro* activity against six clinical isolates of* Helicobacter pylori*, with MICs between 1–8 and 60–120 mg/L, respectively [78].

### 2.4. Antimicrobial Activity

#### 2.4.1. Antiparasitic

Artemisinin and its analogues show marked activity against* Plasmodium* species* in vivo* and* in vitro*. It is effective even against multidrug resistant strains of the malaria parasite and in cases of cerebral malaria. Nowadays, artemisinin and its derivatives are recommended by the World Health Organisation to be used as first choice therapy in the treatment of malaria as part of ACT (artemisinin combination therapy).

Artemisinin has an endoperoxide bridge to which its antimalarial properties are attributed. The proposed mechanism of action involves the formation of free-radical intermediates, resulting from the direct interaction of the endoperoxide group with the intraparasitic iron, and the alkylation of malarial-specific proteins by the artemisinin-derived free radicals, thus damaging the microorganelles and membranes of the parasite. This radical will damage the infected blood cell, which will lead to the disposal of the cell by the hosts own immune system [79]. Artemisinin also targets the parasite mitochondria or the translationally controlled tumour protein and PfATP6, a parasite-encoded sarcoplasmic-endoplasmic reticulum calcium ATPase, which is crucial for the development of the parasite [80].

Other sesquiterpene lactones isolated from* Artemisia* species also showed antimalarial properties. From the leaves and flowers of* Artemisia gorgonum* several sesquiterpene lactones were isolated and evaluated for antiplasmodial activity. Compounds ridentin and hanphyllin had an inhibitory concentration 50 (IC_50_) of 5.4 and 2.3 *μ*g/mL against* Plasmodium falciparum*, respectively. The antimalarial activity may be attributed to the exomethylene group of the lactone function [81].

Dihydroartemisinin, the main metabolite of artemisinin, is a broad-spectrum antiparasitic drug, being active against* Plasmodium, Schistosoma, Toxoplasma, Trichomonas vaginalis, Leishmania,* and* Giardia lamblia* [82].

Dehydroleucodine induces programmed cell death in both the replicative epimastigote form and the infective trypomastigote form of* Trypanosoma cruzi*, which is a different mechanism of action than the conventional drugs to kill the parasite. A combination of DhL with conventional antichagasic drugs showed synergic activity on decreasing parasite viability. Chagas disease or American Trypanosomiasis is caused by the flagellated protozoan parasite* Trypanosoma cruzi* and is one of the world's neglected tropical diseases [83].

Visceral leishmaniasis, caused by the protozoan* Leishmania* sp., affects 500,000 people annually and emerging resistance to conventional antimony therapy has underlined the need for safer yet effective antileishmanial drugs. Artemisinin exhibited antipromastigote activity with IC_50_ ranging from 100 to 120 *μ*M in* Leishmania donovani*,* Leishmania infantum*,* Leishmania tropica*,* Leishmania mexicana*,* Leishmania amazonensis,* and* Leishmania braziliensis*. It was demonstrated that artemisinin exerted a direct parasiticidal activity, while also inducing a host protective response. For* in vivo* studies, the BALB/c mouse model meets eligibility requirements such as the chronic infection pattern, which resembles human visceral leishmaniasis. In* in vivo* studies on mouse model, treatment with artemisinin led to a significant reduction in splenic weight, a significant inhibition of parasites and a restoration of cytokines such as interferon-*γ* and interleukin-2 (IL-2) [84].

Santonin, a sesquiterpene lactone isolated from* Artemisia cina* or other santonin-containing species of* Artemisia,* was widely used in the past as an anthelminthic, a drug that expels parasitic worms from the body, by either killing or stunning them. Due to the severe side effects, the need for a purgative, and the development of many safer anthelmintic drugs, santonin has largely fallen out of use [85].

#### 2.4.2. Antibacterial

Sesquiterpene lactones are one of the main mechanisms of plants defense against microbial attacks. They act by disruption of a microbe's cell membrane, an effect attributable to the polar groups on these antimicrobial compounds disrupting the phospholipid membrane [2].

In an attempt to isolate antibacterial constituents from* Artemisia princeps* var.* orientalis*, secotanapartholides A and B were identified as bioactive compounds. These sesquiterpene lactones produced a clear inhibitory effect against* Clostridium perfringens, Bacteroides fragilis,* and* Staphylococcus aureus* and had no effect on the growth of lactic acid-producing bacteria (*Bifidobacterium adolescentis, Bif. breve, Lactobacillus acidophilus,* and* Lact. casei*) and* Escherichia coli* [86].

Vulgarone B, a component of* Artemisia iwayomogi* essential oil, exhibited significant inhibitory activity against some antibiotic-susceptible and antibiotic-resistant human pathogens. Furthermore, the combination with oxacillin resulted in synergism against antibiotic-resistant* Staphylococcus aureus*. The antibiotic mechanism may involve bacterial DNA cleavage [87].

As mentioned earlier, artemisinin and dehydroleucodine show strong antimicrobial activity against* Helicobacter pylori*, the major cause of chronic gastritis and peptic ulcer [77, 78].

#### 2.4.3. Antifungal

Vulgarone B, a sesquiterpene ketone isolated from the volatile fraction of* Artemisia douglasiana*, exhibited antifungal activity against* Colletotrichum acutatum*,* Colletotrichum fragariae*,* Colletotrichum gloeosporioides,* and* Botrytis cinerea*. Structure-activity studies revealed that the *α*,*β*-unsaturated carbonyl function is a prerequisite for the antifungal activity, so vulgarone B may act as Michael-type acceptor for biological nucleophiles [88].

Artemisinin and its derivatives showed antifungal properties against* Pneumocystis carinii in vitro* [89, 90].

While investigating the action of various sesquiterpene lactones on the growth patterns of four fungal genera,* Colletotrichum*,* Fusarium*,* Botrytis*, and* Phomopsis,* Wedge et al. noticed that the most effective compounds are those that contain an *α*M*γ*L group but lack bulky sterically inhibitory groups, which limit access to the *α*M*γ*L. Also, nonpolar or weakly polar compounds were more bioactive and sesquiterpene lactones of a guaianolide structure had the greatest antifungal potency [91].

#### 2.4.4. Antiviral

Several* in vitro* studies showed that artemisinin has antiviral effect on hepatitis B and C viruses [92, 93], a range of human herpes viruses (human cytomegalovirus, herpes simplex virus type 1, and Epstein-Barr virus) [94–96], influenza virus A [97], and a bovine viral diarrhea virus [98] in the low micromolar range. Artesunate was used successfully for reducing the number of CMV (human herpes virus 5) in an immunosuppressed child without traceable toxicity [99].

## 3. Methods of Analysis of Sesquiterpene Lactones

### 3.1. Extraction and Isolation

The extraction methods of SLs may include common procedures, such as extraction using shaker, sonication process, reflux extraction, or Soxhlet extraction, but also less handy methods, like supercritical fluid extraction (SFE) and microwave-assisted extraction (MAE). The extraction solvents found in the literature are n-hexane, petroleum ether, methanol, acetonitrile, chloroform, toluene, and combinations of them, at different concentrations and different periods of time (from several seconds to days). Isolation of SLs is achieved by further purifying the obtained extracts through repeated column chromatography using different stationary phases (usually normal-phase silica gel) and eluents of increasing polarity (usually hexane-ethyl acetate mixture). The resulting fractions are monitored by TLC in order to separate the compounds of interest. For exemplification, some methods of extraction for well-known SLs in* Artemisia* genus will be described hereafter.

Dehydroleucodine was extracted from* A. douglassiana* after soaking the plant material in chloroform at room temperature, evaporating to dryness and dissolving the extract in ethanol 95%. After removing impurities by treatment with lead tetraacetate solution, the filtrate was extracted three times with chloroform and the resulting extract was chromatographed with ethyl acetate-hexane (1 : 9) to yield dehydroleucodine [71, 100].

Dehydroleucodine and a new SL, named dehydroparishin-B, were identified in the chloroform extract from the aerial parts of* A. douglasiana*. After exhaustively boiling the plant material with chloroform, the extract was partitioned with aqueous 5% NaHCO_3_. The organic phase was subjected to repeated column chromatography on silica gel with hexane-ethyl acetate mixtures to afford dehydroleucodine. The aqueous NaHCO_3_ phase was acidified and extracted with ethyl acetate. The organic phase was evaporated to dryness and subjected to column chromatography, while monitoring the resulting fractions by TLC to give pure dehydroparishin-B [101].

Tehranolide was extracted from* A. diffusa* by maceration 24 hours with a mixture of n-hexane/ethyl acetate/methanol (1 : 1 : 1). The concentrated extract was run through a silica gel column with n-hexane/ethyl acetate mixtures of increasing polarities and further with n-hexane/ethyl acetate/methanol mixtures to produce higher polarities. TLC was used to monitor the fractions and tehranolide was identified by ^13^C-NMR spectra [102].

Yomogin was extracted from the aerial parts of* A. princeps* with methanol at room temperature for 7 days. The concentrated extract was suspended in water and partitioned with dichloromethane and ethyl acetate. Dichloromethane fraction was column chromatographed over silica gel using a gradient elution of methanol and dichloromethane. The bioactive fraction was further purified through repeated column chromatography with hexane and ethyl acetate mixture to give yomogin [60].

Bioactivity guided fractionation was used to isolate yomogin and 1,2,3,4-diepoxy-11(13) eudesmen-12,8-olide from* A. vulgaris* leaves. The plant material was defatted by soaking in hexane for 24 hours, and then the chloroform extract was obtained and chromatographed on a Sephadex LH-20 column with 80% methanol : 20% chloroform. Each subfraction was tested on the guinea pig ileum in order to assess the histamine antagonist activity. The active ones were combined and further purified by repeated preparative TLC, giving a mixture of the two compounds. Alternatively, to increase the yield of active compounds, the crude chloroform extract was defatted repeatedly with petroleum ether and run through silica gel column chromatography using gradient elution of ethyl acetate and dichloromethane to afford a mixture of the same two components. Yomogin was separated through repeated recrystallization with methanol and its structure was confirmed by X-ray crystallography [62].

As a result of impressive hypoglycemic effects* in vivo*, an infusion from the aerial parts of* A. ludoviciana* was subjected to column chromatography in order to identify the active compounds. The dried infusion was partitioned between ethyl acetate and water and the organic phase was chromatographed repeatedly on normal-phase silica gel with ethyl acetate and hexane, yielding six compounds. One of the compounds was the known guaianolide ludartin which manifested significant hypoglycemic effect on its own [103]. Ludartin was also isolated from the crude hexane extract of* A. amygdalina* shoots using a similar procedure [35].

Bioactivity guided fractionation led to the isolation of leucodin from* A. iwayomogi* as moderate antioxidant and antimicrobial compound. The 80% ethanol extract was partitioned successively with n-hexane, chloroform, ethyl acetate, and n-butanol and the fractions were tested for biological activity. The active ethyl acetate-soluble fraction was purified trough repeated column chromatography to yield five compounds, characterized based on EI-MS, UV, IR, and NMR spectral data [104].

Santonin is usually isolated from its primary sources,* A. cina* and* A. maritima*, through chloroform extraction, formation of a barium salt, precipitation of the lactone by acidification, and crystallization from ethanol : water [105]. A new method for santonin extraction from the flowering tops of* A. caerulescens* ssp.* cretacea* involves maceration and percolation over aluminium oxide column with 5% methanol in chloroform. The concentrated extract is exhaustively extracted with boiling water and the aqueous solutions are extracted with chloroform to yield santonin [106]. Santonin was also extracted from aerial parts of* A. pallens* with acetone or acetone : methanol, followed by extract fractionation on silica gel using n-hexane and hexane : acetone [107, 108].

For artemisinin extraction, the most applied technique is liquid solvent extraction with toluene, n-hexane, chloroform, or petroleum ether and extraction times ranging from a few minutes to several hours. The first published laboratory method for artemisinin isolation consisted in the extraction of* A. annua* leaves with petroleum ether followed by column chromatography of the extract over silica gel and elution with a chloroform-ethyl acetate mixture [109].

Artemisinin and its precursors, arteannuin B and artemisinic acid, were isolated from* A. annua* leaves after 100% ethanol extraction at room temperature and fractionation with ethyl acetate and column chromatography of ethyl acetate phase on silica gel with a mixture of petroleum ether and ethyl acetate of increasing polarity. The fractions were monitored through TLC, combined and purified by crystallization to afford the SLs [54].

Rhianna Briars and Larysa Paniwnyk compared a conventional method of extraction of artemisinin from* Artemisia annua* leaf with hexane in a water bath at temperature of 25°C, 35°C, and 45°C with an ultrasonic extraction at the same temperature. After HPLC analysis it was observed that ultrasonic extraction at lower temperature is better than at a higher temperature, also improving the purity [110].

An efficient and fast method with low consumption of solvents is the microwave-assisted extraction (MAE) [111]. Extraction of artemisinin by microwave-assisted extraction was performed in a closed vessel apparatus allowing temperature control and programmable heating power. Extractions were carried out with water, ethanol, toluene, or n-hexane, at 60°C temperature, except for hexane (35°C). Artemisinin recovery was similar with ethanol, toluene, and n-hexane. Water extraction did not succeed as the plant extract degraded in this solvent. Optimal extraction conditions were extraction time 12 minutes, vegetal particles diameter 0,125 mm, and solvent/plant ratio higher than 11 [112]. Liu et al. compared four methods of artemisinin extraction from leaves, flower buds, stems, and roots of* Artemisia annua*: an extraction method at room temperature, heat-reflux extraction at 50°C, a Soxhlet extraction at 50°C, and microwave-assisted extraction at the same temperature of 50°C. They demonstrated that after MAE extraction a high recovery of artemisinin is obtained in less time and with less consumption of reagents [113].

Pressurized solvent extraction (PSE) uses conventional solvents at elevated temperatures and pressures which bring about liquid extraction above the boiling point of the solvent. This technique was applied to powdered* Artemisia annua* leaves loaded into an extraction cell and placed in a thermostated oven. The selected extraction solvent (water or ethanol) was pumped through the extraction cell at a flow-rate of 0,5 mL/min for 20 minutes. Pressure had no noticeable influence on the recovery of artemisinin, whatever the solvent used, but a higher temperature significantly favoured artemisinin extraction, particularly in water [114].

In recent years, supercritical fluid extraction (SFE) has become the method of choice for the extraction of secondary metabolites from plant material. Thus, using a supercritical fluid composed of CO_2_ and 3% methanol at 50°C temperature, 15 MPa pressure, and 2 mL/min flow-rate, artemisinin was quantitatively extracted from the aerial parts of the plant. These mild conditions avoid the degradation of the analytes and allowed us to obtain clean plant extract that does not need further purification. By adding 16.25% ethanol as cosolvent to the supercritical fluid extraction with CO_2_, the artemisinin extraction yields were substantially improved [115, 116].

In order to analyze artemisinin and artemisinic acid, Kohler et al. associated supercritical fluid extraction (SFE) with supercritical fluid chromatography (SFC) coupled with flame ionization detector (FID), which allowed the determination of compounds without a precleaning step [117].

Two SLs from* A. princeps*, artecanin and canin, were isolated through chromatographic separation of the methanol extract and identified by MS and NMR data analysis. After partitioning the methanol extract with hexane and dichloromethane, the latter fraction was subjected to repeated column chromatography on silica gel with dichloromethane-methanol and ethyl acetate-hexane mixtures. The selected fraction was further chromatographed on Sephadex LH-20 column with dichloromethane-methanol (1 : 1) and subfractions subjected to flash-chromatography on RP-18 column with methanol-water mixtures to afford artecanin and canin [118].

Using preparative chromatographic techniques, Martins et al. isolated from the chloroform extract of* A. gorgonum* 11 compounds that included SLs arborescin, arglabin, deacetylglobicin, 2*α*-hydroxyarborescin, sanchillin, and hanphyllin. The extract was run over silica gel columns and eluted with ethyl acetate/n-hexane, ethyl acetate/toluene, and dichloromethane/methanol mixtures of increasing polarity [119].

Two new guaianolides were isolated from* A. argyi* leaves after extraction with 95% ethanol and partition of the concentrated extract between petroleum ether and chloroform. The chloroform extract was repeatedly fractioned and the resulting subfractions were purified by semipreparative HPLC to afford artemisinin A and isoartemisolide [120].

### 3.2. Detection and Quantification

#### 3.2.1. UV-Vis Spectrophotometry

When applying a cheap, simple, and handy method to all laboratories, such as spectrophotometry, a problem can occur in case of analytes that do not have specific chromophore groups in molecule [121] and thus have no significant absorption in the UV-Vis work domain and also do not possess specific chemical groups able to react with certain compounds to form colored products [122]. For these reasons, analysis of sesquiterpene lactones through UV-Vis spectrophotometry is not an easy task.

One of the specific methods for determination of artemisinin in UV domain has the next principle: absorbance measurement of a reaction product of artemisinin in strong alkaline solution. The reaction is completed in 15 minutes and the reaction product is stable for 5 hours [123]. For dissolution of artemisinin different solvents can be used like DMSO, methanol, ethanol, ethyl acetate, and sodium hydroxide and as alkaline reagents potassium hydroxide, calcium hydroxide, sodium carbonate, and sodium bicarbonate. The interaction between artemisinin and the alkaline medium produces a homogenous electronic transition band at 250–330 nm, with a maximum absorbance at 291 nm, and the resulting product is monotype, as shown by the Gaussian curve (bell shape curve). All solvents used show similar spectral resolutions, but peak intensity is decreasing in the order: DMSO, methanol, ethanol, and ethyl acetate. Also, the best reactivity was recorded in the case of sodium hydroxide and potassium hydroxide, with the peak transition varying with concentration.

Just like artemisinin, determination of artesunate is challenging because it has not a distinct chromophore and presents a peroxide bridge which absorbs at lower wavelengths [114, 124]. In order to determine artesunate in tablets, a very simple and sensitive method can be used: artesunate tablets are dissolved in simulated intestinal fluid (monobasic potassium phosphate and sodium hydroxide, pH 6.8) and the absorbance is measured. The pH 6.8 protects the basic chemical nucleus without breaking the lactone ring. The maximum reproducible absorbance is reached at 287 nm with good values of detection limit and quantification limit. Excipients do not interfere with the determination [125].

Other methods approach the endoperoxide ring destruction and introduction in the molecule of at least one double bond. For this purpose, a method comprising two steps has been developed and validated: ethanol solution of artesunate was subjected to alkaline hydrolysis with sodium hydroxide at 50 ± 0.1°C for 60 minutes. After cooling, the solution was treated with acetic acid in ethanol, and the reaction product had an absorbance maximum at 242 nm, yielding apparently furanose acetal, which presents conjugated double bonds, a chromophore with UV absorption [126].

Direct determination of artemisinin and its derivatives in the visible domain, by treatment with certain reagents in order to obtain colored compounds, is not possible, because these artemisinins do not have chemical groups that react easily. Thus, it is necessary to have an intermediate step in which by treatment with acids or bases they are transformed into more reactive compounds, such as enolate/carboxylates or *α*,*β*-unsaturated decalones, and then reaction with certain reagents to form colored compounds or to bleach, depending on concentration [122, 127, 128].

Thus, for determination of artemisinin and its derivatives accurate, simple, and fast spectrophotometric methods have been developed and validated. They are based on the cleavage of endoperoxide linkage in acidic medium (hydrochloric acid), releasing hydrogen peroxide (H_2_O_2_), which reacts with potassium iodide and releases iodine in equivalent amount. Further, the released iodine reacts with various chromogenic agents. As chromogenic agent safranin O can be used. A constant and maximum absorbance is obtained in the range of pH 4-5, registered at 521 nm, with the system being stable for a period of 2 hours. There is a bleach of red colored safranin O, which is transformed in leuco form proportional to the concentration of the analyte. In case of artemisinin determination from tablets, the interferences level was considered acceptable and the excipients used did not hinder the determination [129]. The same method can be applied in the same conditions for artesunate determination [130].

Other chromogenic agents used for determination of both artemisinin and artesunate from tablets are methylene blue and soluble starch. In the first case, the released iodine from the same reaction bleaches methylene blue proportional to concentration and the absorbance is recorded at 665.6 nm. In the second case, the released iodine will form a violet colour with starch, the colour is proportional to the concentration of the analyte, and the absorbance is recorded at 445.6 nm. In case of artesunate, the method which uses methylene blue is more sensitive and more selective, and in case of artemisinin the one who uses soluble starch [121].

Determination of artesunate can also be performed using variamine blue as chromogenic agent. The released iodine oxidizes the leuco form of variamine blue and forms a purple colour compound, the colour being proportional to the concentration of the analyte and is recorded at 556 nm.

Another accurate and precise method for determination of artemisinin and its derivatives is based on a decomposition process in acidic medium at elevated temperatures. The resulted compound presents reactive methylene centres that have the ability to quickly release protons and reduce an acidic solution of p-dimethylaminobenzaldehyde to [4-(dimethylamino) phenyl] methanol, at an optimal temperature of 60°C for 25 minutes. The purple colored product is stable for 4 hours in the laboratory environment and has a maximum absorbance at 540 nm, proportional to the analyte concentration [122].

Artesunate analysis can be done through a simple and inexpensive method after alkaline decomposition of the compound and the reaction between decomposition product with a diazonium salt, 1,5-naphthalene disulfonate salt (Fast Red TR salt). The reaction is pH dependent and positive for artesunate at pH 4. A yellow coloration will be obtained, proportional to the concentration of the analyte, with maximum of absorbance at 420 nm [131, 132]. This test cannot be applied for artemether and therefore Green et al. have developed a method to determine artemether, artesunate, and dihydroartemisinin. In that case decomposition was performed in acidic medium, and after an incubation period of 4 hours, *α*,*β*-unsaturated decalone was obtained. This compound was diazo-coupled with the same diazonium Fast Red TR salt, producing a yellow colour in 5 minutes [132].

For analysis of dihydroartemisinin from tablets, a derivatization reaction can be used, after decomposition in acidic medium, at high-temperature (90°C) with formation of a carbonyl compound. The carbonyl compound reacts with p-nitroaniline, yielding a yellow coloured adduct which shows peaks at 205, 230, and 380 nm [133].

In case of artemisinins analysis from tablets, the extraction of interest analytes is unnecessary because the excipients do not influence the methods described until now, in case analysis from plants is necessary to separate them from the vegetal product and then perform spectrophotometric analysis. An example is that of artemisinin extracted with toluene from the vegetal product in [134] and subjected to a process of alkaline hydrolysis at 50°C for 45 minutes. A mixture of ethanol and trifluoroacetic acid was used as solvent for determination of absorbance at 218 nm [135].

Another example of artemisinin analysis from* Artemisia annua* plant implies Soxhlet extraction with petroleum ether and n-hexane prior to derivatization. Derivatization is achieved by treatment with 0.25% NaOH solution at 50°C for 1 and 1.5 hours and then neutralization with acetic acid 0.2 M. UV spectra are recorded at 203 nm prior to and at 258 nm after derivatization. It was observed that after derivatization the extinction coefficient increased 30–40 times [136].

#### 3.2.2. High Performance Liquid Chromatography

HPLC is the most commonly used technique for the quantification of artemisinin and its derivatives in plants, using detection methods like UV detection (HPLC-UV) or diode array detection (HPLC-DAD), mass spectrometry (HPLC-MS), HPLC/tandem mass spectrometry (LC/MS/MS), evaporative light scattering detector (HPLC-ELSD), and electrochemical detection (HPLC-ECD).

The HPLC-UV and HPLC-DAD analysis requires a pre- or postcolumn derivatization [137], process which in case of HPLC-ELSD is not necessary, which means an advantage of the latter method. On the other hand, its sensitivity is lower than other detection methods, such as ECD and MS [113, 138, 139]. A disadvantage of HPLC-ECD is the requirement of elimination of oxygen from system [117]. HPLC analysis of artemisinin and its derivatives is influenced by the extraction method from vegetal products, mobile phase, column, and detector used.

Since artemisinin detection is difficult, in most cases, after extraction the compound is derivatized using NaOH solutions of different concentrations (0.2%, 0.25%), at different temperatures, the optimum being 50°C, for a period of time ranging from 30 minutes to 1 hour, followed by neutralization with acetic acid of different concentrations (0.08 M or 0.2 M).

The HPLC columns used are suitable for determining the sesquiterpene lactones, with most authors using a normal phase C18 or a reversed-phase C18, but also LC-CN column or silica gel RP-60, with different dimensions: length 50–250 mm, 2.1–4.6 mm ID, and 1.8–5 *μ*m particle size. Generally the column temperature is 30°C, but analyses can be conducted at room temperature, too.

As elution methods, both gradient and isocratic elution were used. Mobile phases for isocratic elution consist of methanol : water/sodium phosphate buffer/acetonitrile. In case of gradient elution, the mobile phases contain different combinations: phosphate buffer : acetonitrile/methanol and methanol or water modified with trifluoroacetic acid to adjust the pH to 3.0–3.5 or phosphoric acid-methanol/acetonitrile.

Highly used detection techniques include UV/DAD from 254 to 350 nm depending on the compound, MS positive ESI mode, ELSD, and the association LC-DAD-MS that has a great specificity [140].  Table 1 contains an overview of HPLC methods applied to sesquiterpene lactones analysis in* Artemisia* species. The majority of HPLC analyses described in the literature are for artemisinin and related compounds.

#### 3.2.3. Gas Chromatography

Sesquiterpene lactones analysis by gas chromatography (GC) is difficult because on one hand the majority of them are thermolabile substances and on the other hand they are less volatile compounds. For this reason, derivatization or transformation into stable degradation products is needed. Detection methods applied in sesquiterpene lactones analysis include GC-MS [141], GC-ECD, and GC-FID [139].

For example,* Artemisia pallens* extract was analysed by GC with MS detection (impact ionization), showing the presence of compounds, such as alpha-santonin, diisobutyl phthalate, tetradecane, and hexadecane [107].

Liu et al. have developed and validated a sensitive method of artemisinin quantification by gas chromatography with ECD detection (electron-capture detection), although not analyzing artemisinin as a whole molecule due to its thermal instability. This method uses small quantities of plant (*Artemisia annua*) and intermediate steps in processing the samples such as extraction, centrifugation, and evaporation have been removed. The samples were analyzed directly after a single solvent one-step extraction, with 97% recovery or more and a limit of detection and quantification less than 3 *μ*g/mL and 9 *μ*g/mL, respectively [113].

GC with flame ionization detection was also applied in analysis of SLs. A retention time of 7.57 minutes was recorded for artemisinin, but other peaks were observed at around 2.35 minutes (artemisinic acid), 4.3 minutes (deoxyartemisinin), and 4.59 minutes (arteannuin B) and their identity has to be confirmed by mass spectrometric detection. The analysis of artemisinin by GC-FID is made via major degradation products, unlike HPLC-ELSD [138]. Another GC-FID method applied to determine artemisinin from* Artemisia annua* extract in hexane led to a retention time of 24.6/34,4 minutes and 30.6/32.2 minutes for arteannuin B [142].

#### 3.2.4. Thin Layer Chromatography

For fast and simple analysis, TLC-densitometric technique can be used. This is based on the transformation of artemisinin after treatment with ammonia vapors in a compound containing chromophore group, 10-azadesoxyartemisinin, detected by UV based TLC densitometry [143].

Simultaneously, determination of artemisinin, arteannuin-B, and artemisinic acid at nanograms levels from* Artemisia annua* can be achieved by using RP-18 F_254S_ thin-layer chromatographic plates, mobile phase containing 0.2% trifluoroacetic acid in water/acetonitrile (35 : 65, v/v), derivatization with anisaldehyde reagent in acidic medium, and densitometric determination at 426 nm in absorption-reflectance mode [144].

Widmer et al. developed an extract from* Artemisia annua* leaves (obtained by sonication in toluene) on silica gel 60 plates with mobile phase cyclohexane : ethyl acetate : acetic acid (20 : 10 : 1), derivatization with anisaldehyde reagent in ethanol : water (10 : 8), heated at 100°C, for 12 minutes, and densitometric evaluation of fluorescence at 520 nm [145].

TLC evaluation of artemisinin can be carried out on silica gel RP-18 60 F_254_ plates, with mobile phase methanol : acetonitrile : ethyl acetate : acetic acid (30 : 20 : 2 : 1) and densitometric evaluation at 254 nm. The limits of detection and quantification were 4 *μ*g/mL and 10 *μ*g/mL, respectively.

Rimada et al. chose for TLC analysis silica gel with fluorescent indicator, at 254 nm, followed by derivatization with 1% vanillin in sulphuric acid, at 105–110°C. The results demonstrated the presence of arteannuin B, artemisinin, and artemisitone [142].

In order to analyze santonin, an accurate, reproducible, simple, and rapid high performance thin layer chromatography method (HPTLC) has been developed. Precoated aluminum plates with silica gel 60 F_254_ were used, mobile phase hexanes : ethyl acetate (3 : 2), and densitometric detection at 258 nm [108].

#### 3.2.5. Other Methods of Analysis

Another method used to analyze artemisinin and artemisinic acid is supercritical fluid chromatography (SFC) coupled with flame ionization detector (FID) [117].

Reys et al. developed a feasible alternative method that uses an amperometric detector based on hemin adsorbed on silica gel modified for quantification of artemisinin [146].

Also, ^1^HNMR is a suitable and valid method applied by Rimada et al. for artemisinin analysis in purified* Artemisia annua* extract in presence of N, N-dimethyl-formamide as internal standard [142].

### 3.3. Structure Identification

The complete elucidation of a compound structure by a single method is an impossible mission, this being achieved by a combination of several methods of analysis, among which IR, NMR, and MS.

In IR spectroscopy, due to the complexity of the spectra, specific bands are attributable accurately only when these are intense and correspond to groups such as carbonyl, hydroxyl, C–H, and aromatic rings. It is important to remember that there are no two different substances with the same IR spectrum, especially when using the area under the 1500 cm^−1^, which is considered the fingerprinting area.

Through magnetic resonance imaging the following characteristics can be established: the structural data of the organic compounds, the dynamic properties of the molecules, the quantitative analysis of the compounds as such or in mixtures, the percentage of hydrogen in an unknown sample, the number and type of carbon atoms in the structure, and the position of carbon atoms, with or without protons.

Mass spectroscopy is an instrumental method of analysis which is based on the fragmentation of the molecules of organic substances by radiation with high energy, up to 100 eV, and the analysis of the number, the charge, and mass of the resulting fragments to obtain information on the structure and identity of researched substances. Due to energy accumulation, fragmentation of molecules occurs with breaking of interatomic bonds, a process resulting mostly in positive ions (seldom negative), radicals, radical ions, and neutral molecules. These fragments constitute important parts in the recreation of the molecular structure.

**Figure 2:**
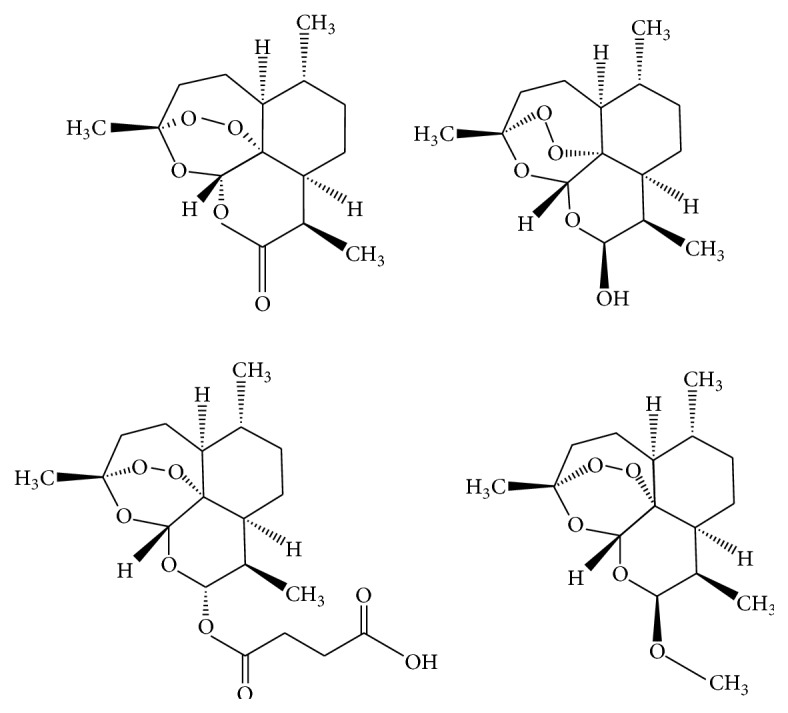
Artemisinin, dihydroartemisinin, artesunate, and artemether.

Next, the spectra of well-known sesquiterpene lactones from* Artemisia* genus are discussed comparatively : artemisinin, dihydroartemisinin, artesunate, and artemether (Figure 2). Artesunate is obtained from the reduction of artemisinin to dihydroartemisinin and esterification of the latter with succinic anhydride and artemether is obtained by treatment of dihydroartemisinin with methanol and an acid catalyst [147].

In the case of IR spectra, the common elements are attributed to stretching vibrations of C=O (1420–1300 cm^−1^), C–O (1380–1370 cm^−1^, 1235 cm^−1^, and 1093 cm^−1^), C–O–O–C (890–820 cm^−1^, 1121.62 cm^−1^), O–O (825 cm^−1^), C–O–C (1023.89 cm^−1^ and 1277.83 cm^−1^), C–H bending vibrations (1225–950 cm^−1^), C–H stretching vibrations (2844.99, 2873.61, 2914.58, and 2936.97 cm^−1^), rocking vibration of CH_2_ (700 cm^−1^) and CH_3_ (2947 cm^−1^), and aromatic ring vibrations (1650–1400 cm^−1^ and 2000–1620 cm^−1^) [121, 148, 149].

Differences occur in the region 1750–1725 cm^−1^ and 1005–925 cm^−1^, for vibrations of C–O–C=O and CH_2_–CH_2_ bonds in artesunate and the appearance of vibrations that prove transformation of C=O in C–O (1034.14 cm^−1^) and the presence of OH group (3371.57 cm^−1^) in dihydroartemisinin [148].

The spectrum of artemisinin and dihydroartemisinin contains each 15 carbon atoms, which consists of 3 methyl groups (CH_3_) 4 methylene groups (CH_2_), 5 methine groups (CH), and 3 quaternary carbon atoms [148]. The artemether spectrum contains 16 carbon atoms, having an extra methyl group (CH_3_) in position 10, and artesunate contains 19 carbon atoms, with the addition of a succinyl group in position 10.

For the methyl groups in positions 3, 6, and 9, the chemical shifts in ^13^C NMR spectrum recorded the values 13.37, 20.56, and 26.26 ppm. For C-10 atom, if artemisinin corresponds to a chemical shift of 172.24 ppm, the transformation of carbonyl group in OH group is demonstrated by a change in the value to 96.60 ppm [148].

The ^1^H NMR spectra showed the presence of the hydroxyl group at 2.77 ppm (singlet 1H). There were also observed chemical shifts for the protons of the methyl groups at positions 3, 6, and 9 (singlet, 1.43 ppm, and 0.96 ppm doublet) and of methylene groups in positions 4, 5, 7, and 8, multiplet signal.

**Figure 3:**
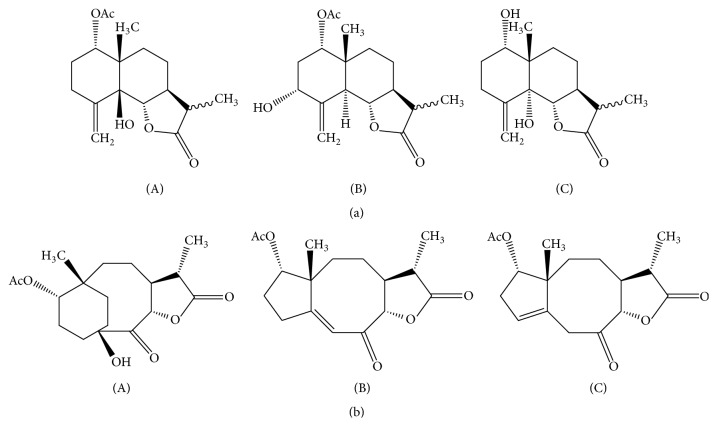
Artemivestinolides (A)–(C) (a) and arvestolide (A)–(C) (b) from* A. vestita.*

Another example of structure elucidation is illustrated by the work of Tian et al. who identified three new eudesmane sesquiterpene lactones called artemivestinolides A-C [150] and three rare sesquiterpenes called arvestolide A-C [151] from* Artemisia vestita* (Figure 3).

The IR spectra of the 6 compounds highlight the presence of OH group in artemivestinolides and arvestolide A at 3478 cm^−1^ and 3354 cm^−1^, respectively. The carbonyl group is present in all cases at 1766, 1726 cm^−1^/1783, 1733 cm^−1^/1773, 1734 cm^−1^/1778, and 1734 cm^−1^. The double bond is only observed in arvestolides B and C, at 1641 and 1673 cm^−1^.

In the ^1^H NMR analysis, the 6 compounds presented signals corresponding to a secondary methyl group (*δ*_H_ 1.20/1.21/1.22/1.27/1.26/1.26), H-13; 2 tertiary methyl groups (*δ*_H_ 1.12/0.88/0.89/0.95/1.31/1.13), H-14, and (*δ*_H_ 2.04/2.13/-/2.08/2.05/2.01), H-17; 2 oxymethine protons (*δ*_H_ 5.13/4.71/4.15/4.69/4.97/5.10), H-1, and (*δ*_H_ 4.60/4.23/4.49/5.13/5.02/4.68), H-6; 2 olefinic protons in artemivestinolide (*δ*_H_ 5.10/5.20/5.03), H-15; and just 1 olefinic proton in both arvestolides B and C (*δ*_H_ 6.11/3.27), H-15.

In the case of ^13^C NMR analysis, all six compounds, with the exception of artemivestinolide C (15 carbon atoms), have 17 carbon atoms, the skeleton being of type 6/6/3 for artemivestinolides, 6/8/3 for arvestolide A, and 5/8/3 for arvestolides B and C. In all compounds, the lactone carbonyl is highlighted, *δ*_C_ 179.1/179.7/180/177.13/176.7/186.4. In five compounds, the acetyl group was observed at C1 (*δ*_C_ 170.6/170.5) and for arvestolide A, the OH group at C4 (*δ*_C_ 73.7) [150, 151].

In the case of MS analysis, generally using ESI positive ionisation mode, the pseudo-molecular ions are in the form [M + Na]^+^, [M + H]^+^, [2M + Na]^+^, [2M + K]^+^, and [M + NH4]^+^ [148, 152]. For example, [M + Na]^+^ is observed in case of arvestolide A* m/z* 347.1465 [151] and artemisinin* m/z* 305 [153], [M + H]^+^ in case of artecanin* m/z* 279.1235 [118].

## 4. Conclusions

Sesquiterpene lactones are large and structurally divers group of natural products, found almost ubiquitously in plants of* Asteraceae* family. Genus* Artemisia*, one of the largest in this family and with worldwide distribution, contains numerous valuable sesquiterpene lactones. They present a broad spectrum of biological activities, such as antitumor, antimalarial, anti-inflammatory, immunomodulatory, antiulcerogenic, antibacterial, antifungal, and antiviral. In most cases, the documented mechanism of action involves the presence of *α-*methylene-*γ-*lactones and *α*,*β*-unsaturated cyclopentenone ring. The present paper proposes an overview of biological activities and of methods used for the identification and quantification of sesquiterpene lactones found in* Artemisia* genus. The potential for drug development from* Artemisia* species continues to grow, particularly in the area of parasitic diseases and cancer treatment. The information summarized here is intended to serve as a reference tool to people in all fields of natural products chemistry.

## Figures and Tables

**Table 1:** Summary of HPLC conditions for sesquiterpene lactones.

Sample	Extraction, derivatization	Detected sesquiterpene lactones	Detection	Chromatographic conditions	Reference
Sandy, clayey, and humic soil	(i) Supercritical fluid extraction (SFE) (ii) Supercritical fluid: CO_2_(iii) Extraction time: 20 minutes (iv) Precolumn derivatization (0.2% NaOH, 50°C, 30 min, then acidified 0.08 M acetic acid)	Artemisinin	UV, 260 nm	(i) Column: C18 Bio Wide Pore (25 cm × 4.6 mm, 5 *μ*m) (ii) Column temperature: 30°C (iii) Elution type: isocratic (iv) The mobile phase: methanol/acetonitrile/0.9 mM Na_2_HPO_4_–3.6 mM NaH_2_PO_4_ buffer (pH 7.76) solution (45/10/45 v/v/v) (v) Injection volume: 20 *μ*L (vi) Flow rate: 1 mL/min (vii) Retention time: 7.5 min	[154]

*A. santonicum *L., *A. taurica *Willd., *A. spicigera *K. Koch, * A. herba-alba *Asso, * A. haussknechtii *Boiss., *A. campestris *L., *A. araratica *Krasch., *A. armeniaca *Lam., *A. austriaca *Jacq., and *A. abrotanum *L.	(i) Extraction with n-hexane at room temperature for 2 days with a laboratory-scale shaker (ii) Precolumn derivatization (0.2% NaOH, 50°C, 30 min, then acidified 0.08 M acetic acid)	Artemisinin	DAD, 254 nm	(i) Column: ACE-5 C18 column (250 × 4.6 mm, 5 *μ*m) (ii) Column temperature: 30°C (iii) Elution type: isocratic (iv) The mobile phase: formic acid (0.2% v/v) : acetonitrile (50 : 50 v/v) (v) Flow rate: 1 mL/min. (vi) Retention time: 5.58 min	[155]

*Artemisia annua* L	(i) Extraction in Soxhlet extractor with petroleum ether (30–60°C) for 6 h (ii) Precolumn derivatization (0.2% NaOH, 45°C, 30 min, and then acidified 0.08 M acetic acid)	Artemisinin	UV, 260 nm	(i) Column: RP-C18 silica column (250 × 4.6 mm, 5 *μ*m) (ii) Column temperature: 30°C (iii) Elution type: isocratic (iv) The mobile phase: methanol/acetonitrile/0.9 mM Na_2_HPO_4_–3.6 mM NaH_2_PO_4_ buffer (pH 7.76) solution (45/10/45 v/v/v) (v) Injection volume: 10 *μ*L (vi) Flow rate: 0.5 mL/min (vii) Retention time: 16.85 min	[156]

*A. absinthium *leaves	(i) Extraction with various solvent types: 100% methanol, 75% methanol, 50% methanol, 25% methanol, 75% acetonitrile in a thermostatic rotary shaker, and various temperatures (30–60°C) for various time intervals (ii) Precolumn derivatization (0.25% NaOH, 50°C, 1 hour and then being acidified with 0.2 M acetic acid solution)	Anabsinthin and derivatized artemisinin	DAD, 205 and 258 nm	(i) Column: C8 (250 mm × 4.6 mm, 5 *μ*m) (ii) Column temperature: 30°C (iii) Flow rate: 1 mL/min (iv) Elution type: gradient elution: 90% A/10% B, hold for 5 min, to 60% B in the next 13 min (v) The mobile phase: A: methanol/0.1% trifluoroacetic acid (TFA) (15/85), B: methanol/0.1% TFA (85/15) (vi) Artemisinin not detected	[157]

*A. annua* leaves	(i) Dipping dried leaves in 100% chloroform, 8 s (ii) Precolumn derivatization (0.2% NaOH and then acidified with 0.08 M acetic acid solution)	Artemisinin	260 nm	(i) Column: RP-C18 silica column (250 × 4.6 mm) (ii) Elution type: isocratic (iii) The mobile phase: methanol/acetonitrile/0.9 mM Na_2_HPO_4_–3.6 mM NaH_2_PO_4_ buffer (pH 7.76) solution (45/10/45 v/v/v) (iv) injection volume: 1 *μ*L	[158]

*A. annua* leaves	(i) Solid phase extraction (ii) Liquid-liquid extraction method (iii) Purification procedures	Artemisinin	IR	(i) Column: LC-CN column (25 mm × 4 mm × 5 *μ*m) (ii) Column temperature: 35°C (iii) Elution type: isocratic (iv) The mobile phase: methanol : water (60 : 40 v/v) (v) Injection volume: 20 *μ*L (vi) Flow rate: of 1 mL/min (vii) Retention time: 6.932 min	[159]

*A. annua*	Extraction by refluxing with hexane at 75°C for 1 hour	Artemisinin	ELSD	(i) Column: C18-RP (250 mm × 4.0 mm, 5 *μ*m) (ii) Column temperature: room temperature (iii) Elution type: isocratic (iv) The mobile phase: water adjusted to pH 3.0–3.5 with trifluoroacetic acid (TFA) : acetonitrile (65 : 35) (v) Flow rate: 1.0 mL/min (vi) Retention time: 7.63 min	[138]

*A. herba alba *and *A. monosperma* aerial parts	Extraction in Soxhlet extractor with methanol at 60°C	*α*, *β*-Dihydroartemisinin, dihydroartemisinic aldehyde, arteannuin B, dihydroartemisinic acid, dihydroartemisinic alcohol, artemisitene, artemisinin, and artemisinic acid	HPLC-DAD (215, 254, 294, and 334 nm), LC-positive mode ESI-MSn	(i) Column: C18 column (50 mm × 2.1 mm, 1.8 *μ*m) (ii) Column temperature: 30°C (iii) Elution type: gradient, 0 min—A : B 10 : 90; 36 min—A : B 100 : 0; 40 min—A : B 100 : 0 (iv) The mobile phase: A: methanol, B: 0.2% formic acid (v) Injection volume: 10 *μ*L (vi) Flow rate: 0.2 mL/min (vii) Retention time: 12.4 min *α*-dihydroartemisinin, 12.8 min *β*-dihydroartemisinin, 15.2 min dihydroartemisinic aldehyde, 15.5 min arteannuin B, 15.7 min dihydroartemisinic acid, 18.8 min dihydroartemisinic alcohol, 36.6 min artemisitene, 37.7 min artemisinin, and 23.7 min artemisinic acid	[160]

*A. annua*	(i) Extracted twice with scintanalyzed toluene in a ultrasonic bath, in ice-cold water, for 30 minutes (ii) Precolumn derivatization	Artemisinin	260 nm	(i) Column: C-18 column (15 cm × 4.6 mm, 5 *μ*m) (ii) Elution type: isocratic (iii) The mobile phase: 0.01 M sodium phosphate buffer: methanol [55 : 45 (v/v)] pH 7.0 (iv) Flow rate: 1 mL/min (v) Retention time: 12.0 min	[161]

*A. annua* leaves	Extraction in Soxhlet extractor with petroleum ether : n-hexane (2 : 1) for 4 hours	Artemisinin	DAD, 258 nm	(i) Column: C8 (250 × 4.6 mm, 5 *μ*m) (ii) Column temperature: 30°C (iii) Elution type: gradient, 5 min—70% A: 30% B to 60% B in the next 13 min (iv) The mobile phase: A: 0.9 mM Na_2_HPO_4_, 3.6 mM NaH_2_PO_4_ buffer (pH 7.76); B: acetonitrile (v) Injection volume: 20 *μ*L (vi) Flow rate: 1 mL/min (vii) Retention time: 6.476 min	[136]

*A. annua* leaves	(i) Sonication with toluene (ii) Precolumn derivatization (0.2% NaOH, 50°C, 45 min and then acidified 0.2 M acetic acid)	Artemisinin	DAD, 258 nm	(i) Column SB C18 column (150 × 4.6 mm) 5 *μ*m (ii) Elution type: isocratic (iii) The mobile phase: 45% (v/v) methanol and 55% 0.01 M sodium phosphate buffer (pH7.0) (iv) Flow rate: 1 mL/min (v) Retention time: 12 min	[162]

*A. annua *L leaves, flower buds, stems, and roots	(i) Room temperature extraction (ii) Heat-reflux extraction at 50°C (iii) Soxhlet extraction at 50°C (iv) MAE (microwave-assisted extraction) (v) Solvent: petroleum ether : acetone (4 : 1, v/v)	Artemisinin	ELSD	(i) Column: RP-C18 column (150 mm × 4.6 mm i.d., 5 *μ*m) (ii) Column temperature: 30°C (iii) Elution type: isocratic (iv) The mobile phase: water : acetonitrile (40 : 60 v/v) (v) Injection volume: 10 *μ*L (vi) Flow rate: 1 mL/min (vii) Retention time: 9 min	[113]

*A. annua *L	Extraction with methanol by sonication, 45 minutes	Artemisinin	LC-MS with SIM	(i) Column: ODS3 column (250 × 4.6 mm, 5 *μ*m) (ii) Elution type: gradient, 72% B for 6 min, and it was then increased to 100% B in 1 min (iii) The mobile phase: water (0.1% formic acid) and (B) acetonitrile (iv) Injection volume: 2 *μ*L (v) Flow rate: 1.2 mL/min (vi) *m*/*z* 265.3	[163]

*A. annua *seeds, aerial parts	Extraction in Soxhlet extractor with methanol, 60°C	Artemisinin	HPLC/DAD 214, 217, 280, and 290 nm HPLC-positive mode ESI-MS	(i) Column: SB-C18 column (150 mm × 4.6 mm i.d., 1.8 *μ*m) (ii) Column temperature: 30°C (iii) Elution type: gradient, 0 min, A : B 10 : 90; 36 min, A : B 70 : 30; 50 min, A : B 100 : 0; 60 min (iv) The mobile phase: (A) methanol and (B) 0.2% formic acid (v) Flow rate: 0.8 mL/min (vi) Retention time: 35.2 min	[140]

*A. annua *L	Maceration with dichloromethane or hexane, at room temperature, for 72 hours	Artemisinin	HPLC-DAD (210 nm), HPLC-MS (API electrospray)	(i) Column: RP-18 column (250 mm × 4.6 mm i.d., 5 *μ*m) (ii) Column temperature: 26°C (iii) Elution type: isocratic (iv) The mobile phase: water adjusted to pH 3.2 by formic acid (A), and acetonitrile (B) 50% A : 50% B (v) Injection volume: 20 *μ*L (vi) Flow rate: 1.3 mL/min (vii) Retention time: 15.1 min	[153]

*A. annua *L	Extraction with different solvents and mixtures: n-hexane, isopropyl alcohol, ethanol, toluene by maceration, percolation, or decoction, at low temperatures and vigorous shaking	Artemisinin, arteannuin, and artemisitone	RP-HPLC/refraction index	(i) Column: RP-C18 column (100 mm × 4 mm i.d., 3 *μ*m) (ii) Column temperature: room temperature (iii) Elution type: isocratic (iv) The mobile phase: methanol (80–90%), water (v) Injection volume: 100 *μ*L (vi) Flow rate: 0.5 mL/min (vii) Retention time: 1.7 min (arteannuin), 2 min (artemisinin), and 5.3 min (artemisitone)	[142]
